# Electron density and thermal motion of diamond at elevated temperatures

**DOI:** 10.1107/S2053273322010154

**Published:** 2023-01-01

**Authors:** Jonas Beyer, Thomas Bjørn Egede Grønbech, Jiawei Zhang, Kenichi Kato, Bo Brummerstedt Iversen

**Affiliations:** a Center for Integrated Materials Research, Department of Chemistry and iNANO, Aarhus University, Langelandsgade 140, 8000 Aarhus C, Denmark; b RIKEN SPring-8 Center, 1-1-1 Kouto, Sayo-cho, Sayo-gun, Hyogo 679-5148, Japan; c JST, PRESTO, 4-1-8 Honcho, Kawaguchi, Saitama 332-0012, Japan; University of Warsaw, Poland

**Keywords:** X-ray electron density, synchrotron powder X-ray diffraction, diamond, convolution approximation

## Abstract

The electron density and thermal motion of diamond are determined at nine temperatures between 100 K and 1000 K via synchrotron powder X-ray diffraction (PXRD) data collected on a high-accuracy detector system. Decoupling of the thermal motion from the thermally smeared electron density is performed via an iterative Wilson–Hansen–Coppens–Rietveld procedure using theoretical static structure factors from density functional theory (DFT) calculations. The thermal motion is found to be harmonic and isotropic in the explored temperature range, and excellent agreement is observed between experimental atomic displacement parameters (ADPs) and those obtained via theoretical harmonic phonon calculations (HPC), even at 1000 K. The Debye temperature of diamond is determined experimentally to be Θ_D_ = 1883 (35) K. A topological analysis of the electron density explores the temperature dependency of the electron density at the bond critical point. The properties are found to be constant throughout the temperature range. The robustness of the electron density confirms the validity of the crystallographic convolution approximation for diamond in the explored temperature range.

## Abstract 2

The electron densities and atomic displacement parameters of diamond are determined from 100 K to 1000 K using synchrotron powder X-ray diffraction.

## 1. Introduction

Diamond is considered by materials scientists to be one of the most well behaved solids due to its high cubic symmetry and simple chemical bonding scheme. The structure consists of carbon atoms bound to each other in a rigid network, leading to interesting properties such as extraordinary hardness (Brookes & Brookes, 1991 ▸) and high thermal conductivity (Olson *et al.*, 1993 ▸). Diamond also holds a special position within the field of X-ray crystallography due to the chemistry of carbon. A neutral carbon atom consists of six electrons, two in the inner shell classified as core electrons and four in the outer shell classified as valence electrons. The single 2*s* and three 2*p* valence orbitals can hybridize in the renowned *sp* ^3^ hybridization with a tetrahedral point symmetry. This hybridization readily forms the covalently bonded network structure, where each carbon atom shares electrons with four others in a tetrahedrally coordinated network of cubic symmetry (space group No. 227, 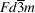 ).

To a first approximation, X-ray diffraction data from crystalline materials are analysed under the assumptions of the independent atom model (IAM). Here, the electron densities (EDs) around atoms are assumed to be rigid and spherically symmetric. However, for accurate modelling of the X-ray scattering from diamond, the IAM model is inadequate due to the non-negligible ED in the covalent bonds. The ED is aspherically deformed into the bonds, which is especially evident in diamond because of the high fraction of electrons taking part in the bonds. This effectively lowers the symmetry, which causes the emergence of the ‘forbidden’ *h* + *k* + *l* = 4*n* + 2 reflections (Coppens, 1997 ▸).

Diamond has already been studied extensively via a variety of techniques (Stewart, 1973 ▸; Post, 1976 ▸; Price *et al.*, 1978 ▸; Williams *et al.*, 1990 ▸; Weidner *et al.*, 1994 ▸; Prawer & Nemanich, 2004 ▸) and theoretical calculations (Chadi & Cohen, 1975 ▸; Stoll, 1992 ▸; Kresse *et al.*, 1995 ▸; Gali *et al.*, 2008 ▸), and the motivation for conducting the additional analysis in this study is twofold. The first is to corroborate the viability of powder X-ray diffraction (PXRD) for studying the ED in crystalline materials. Historically, the preferred technique for ED diffraction measurements has been single-crystal X-ray diffraction (SCXRD). In the last decade, however, PXRD carried out on dedicated synchrotron beamlines using state-of-the-art detectors (Bergamaschi *et al.*, 2010 ▸; Dippel *et al.*, 2015 ▸; Kato *et al.*, 2019 ▸) has been shown to achieve a structural accuracy on a par with SCXRD (Svane *et al.*, 2019 ▸) and, in a recent study, ED modelling of urea against data collected on a MYTHEN micro-strip detector system, corrected for X-ray response non-uniformity to restore the dynamic range (Kato *et al.*, 2019 ▸; Kato & Shigeta, 2020 ▸), was found to reproduce the results found from SCXRD (Svane *et al.*, 2021 ▸). Although SCXRD generally offers higher data quality due to redundant measurement of the crystallographic structure factors, PXRD has several experimental benefits over SCXRD. These include shorter data collection times, minimized sample preparation, reduced absorption and minimization of multiple scattering effects. The latter effect actually posed a significant challenge for early SCXRD studies of diamond since the intensities of the forbidden reflections are significantly affected (Post, 1976 ▸; Coppens, 1997 ▸). For accurate extraction of the structure factors, the main challenge for PXRD compared with SCXRD is peak overlap and treatment of the background intensity (Straasø *et al.*, 2013 ▸; Bindzus *et al.*, 2014 ▸). Fortunately, the peak overlap is minimized for high-symmetry solids with small unit cells, such as diamond, whereby Rietveld modelling (Rietveld, 1969 ▸) can be utilized to partition overlapping reflections properly (Svendsen *et al.*, 2010 ▸). The challenge regarding the background is examined herein and found to only have a minor influence on the analysis for the present data.

The second motivation is to assess the validity of the crystallographic ‘convolution approximation’. A well known challenge for conducting ED studies is decoupling of atomic thermal motion to obtain the static ED. From an experimental point of view, the challenge is that both the thermal motion and the spatial distribution of the ED cause the scattered intensity to diminish at high diffraction vector momentum transfers and thus have a tendency to correlate during model refinement. From a theoretical point of view, it is not obvious that the two are independent. Rigorous motion of the nuclei, which carry most of the atomic mass, could cause the ED to change, especially when constrained by strong chemical interactions such as in diamond. This would result in temperature-dependent static EDs. The convolution approximation makes the assumption that the two can be entirely deconvoluted, *i.e.* that their correlation is solely numerical. It assumes the ED to be completely rigid and centred on the nucleus regardless of thermal motion, resulting in a smearing of the ED as a rigid unit. This very practical assumption allows for separate treatment of the scattering factors and thermal motion during structural modelling and is invoked in the majority of structural X-ray diffraction based studies. Its validity can be assessed by modelling the ED and thermal motion at several temperatures but only if one of them is known. The diamond structure has been reported to have a Debye temperature between 1800 K and 2300 K (Schoening & Vermeulen, 1969 ▸; Zhi-Jian *et al.*, 2009 ▸) and it exhibits only a minor thermal expansion of approximately 1 pm from 0 K to 1250 K (Jacobson & Stoupin, 2019 ▸). In combination, these properties hint at a rigid structure where the ED can be reasonably assumed constant at temperatures from 0 K to 1000 K. In fact, the ED has already been reported to be nearly identical at 300 K and 800 K (Deguchi & Nishibori, 2018 ▸). Diamond therefore serves as a good candidate structure to test the convolution approximation. If the static ED can be shown to be constant with temperature under adequate decoupling of the thermal motion, this would confirm its validity.

The most prevalent method for modelling aspherical deformations of the ED is the Hansen–Coppens (HC) multipole model (Hansen & Coppens, 1978 ▸). Here, the pseudo-atomic ED is described as a sum of core (subscript c) and valence (subscript v) contributions,

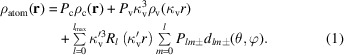

In short, the HC formalism treats the ED of the core electrons as inert and unperturbed (the ‘frozen-core’ approximation), while the valence ED is split into two parts: a spherical and an aspherical term. Both parts are allowed to expand or contract through the κ_v_ and 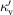 parameters, respectively, while the asphericity is described by the deformation density functions *d*_*lm*±_, which are most often the spherical harmonics. In the case of cubic site symmetry, these are replaced by the Kubic harmonic functions which can be obtained as linear combinations of the spherical harmonics (Kara & Kurki-Suonio, 1981 ▸; Su & Coppens, 1994 ▸). The HC model can also be extended to include asphericity of the core electrons, and the extended HC (EHC) model has been used to characterize the core deformation in diamond and silicon (Fischer *et al.*, 2011 ▸; Bindzus *et al.*, 2014 ▸; Wahlberg *et al.*, 2016 ▸).

Experimental observation and modelling of the ED are cornerstones of X-ray crystallography and this is one of the most direct experimental observations of the quantum mechanics that govern the chemistry of solid materials. Topological analysis of the experimental ED obtained through X-ray diffraction and HC modelling has been used to study a wide range of chemical interactions (Koritsanszky & Coppens, 2001 ▸; Tolborg & Iversen, 2019 ▸), and examples include van der Waals interactions (Kasai *et al.*, 2018 ▸), hydrogen bonds (Tolborg *et al.*, 2019 ▸), intra- and intermolecular interactions (Birkedal *et al.*, 2004 ▸), transition metal bonding (Grønbech *et al.*, 2020 ▸) and *f*-orbital characteristics (Gao *et al.*, 2020 ▸), to name a few.

In this study, decoupling of the thermal motion during HC modelling is achieved in combination with an iterative Wilson procedure, where advantage is taken of the homoatomic nature of diamond (Fischer *et al.*, 2011 ▸; Bindzus *et al.*, 2014 ▸). By assuming that the thermal motion is harmonic and isotropic, the corresponding effect on the structure factors can be described by the Debye–Waller factor *T*_0_ = exp(−8π^2^ *U*_iso_sin^2^(θ)/λ^2^), where *U*_iso_ is the isotropic atomic displacement parameter (ADP). The following relationship between the static structure factors *F*_stat_ [obtained through theoretical computation at experimental geometry using density functional theory (DFT), for instance] and the observed structure factor *F*_obs_ can then be established,

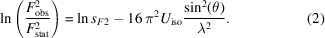

Here, *s*_*F*2_ is a scale factor, such that 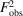 = 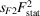 (Giacovazzo *et al.*, 2011 ▸). From equation (2), which is often depicted in a Wilson plot, *i.e.* 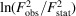 plotted against sin^2^(θ)/λ^2^, it is evident that the ADP governs the slope. This can thus be extracted by a linear least-squares regression. The obtained value of *U*_iso_ from an initial set of extracted *F*_obs_ via the Hansen–Coppens–Rietveld model can be used as a fixed parameter for subsequent refinement to extract a new set of *F*_obs_. This comprises the iterative Wilson–Hansen–Coppens–Rietveld (WHCR) procedure, which should be repeated until *U*_iso_ converges (Bindzus *et al.*, 2014 ▸). The accuracy of this procedure is enhanced by increasing the resolution sin(θ)/λ since a larger number of structure factors can be included in the Wilson plot. In this study, the models were refined against diffraction data collected up to sin(θ)/λ = 1.67 Å^−1^. The WHCR procedure is especially powerful for decoupling thermal effects from the core electrons (Fischer *et al.*, 2011 ▸; Bindzus *et al.*, 2014 ▸; Wahlberg *et al.*, 2016 ▸). However, the core deformation is not taken into account in the present study.

After decoupling of the thermal motion, the resulting set of *F*_obs_ are used to compute the static ED, which is subsequently subjected to a topological analysis according to Bader’s quantum theory of atoms in molecules (QTAIM) (Bader, 1994 ▸). Herein, this is carried out via the *XD2016* software (Volkov *et al.*, 2016 ▸), where the extracted *F*_obs_ are essentially treated as SCXRD structure factors. This prevents correlations with the peak profile and background parameters of the whole-pattern PXRD model, reduces the number of parameters in the final HC model and, consequently, enhances the sensitivity to information in the structure factors (Bindzus *et al.*, 2014 ▸). Under QTAIM, chemical significance is given to critical points in the ED, which are maxima in two directions and minima in one. These are known as bond critical points (BCPs) and are used to quantify chemical bonds in density-based analyses. In diamond, there is only one unique BCP (Bindzus *et al.*, 2014 ▸; Svane *et al.*, 2021 ▸) and it is used herein to quantify differences between the refined density models at different temperatures. Specifically, the density and Laplacian (second derivative of the density) at the BCP are compared between the different temperatures.

## 2. Experimental

### 2.1. Data collection

Diamond powder purchased from Sigma–Aldrich (particle size ≤1 µm) was packed in a quartz capillary with an inner diameter of 0.2 mm and sealed under an argon (Ar) atmosphere in a glove box. PXRD data were collected on the OHGI detector (Kato *et al.*, 2019 ▸) of the RIKEN Materials Science I beamline BL44B2 at the SPring-8 Synchrotron Facility in Hyogo, Japan (Kato *et al.*, 2010 ▸). The energy of the incident X-rays was determined by Le Bail modelling of data from a NIST standard reference material LaB_6_ (SRM660b; Black *et al.*, 2011 ▸) data set to 25.298 (6) keV [λ = 0.49010 (1) Å]. The energy threshold of the detector was set to 12.6 keV, corresponding to approximately half of the incident X-ray energy. Correction factors for X-ray response non-uniformity were collected using the *ReLiEf* algorithm (Kato *et al.*, 2019 ▸; Kato & Shigeta, 2020 ▸). The dimensions of the incident beam were 1 × 0.5 mm (horizontal × vertical). The X-rays from the bending magnet source were assumed completely polarized in the horizontal plane.

Data were collected using the high-resolution strategy with a resolution of 0.005° up to 155.7° in 2θ, corresponding to sin(θ)/λ = 1.99 Å^−1^ (*q* = 25.0 Å^−1^). The temperature was set at points in a range from 100 K to 1000 K in 100 K steps. The sample was heated at a rate of *ca* 100 K min^−1^ and a 2 min waiting period was employed before each measurement for temperature stabilization. Data were collected twice at 300 K: once before heating and once after (the latter is referred to as ‘300 K-after’). The heater was simply shut off when cooling from 1000 K to 300 K, leading to a high but unknown cooling rate. The actual temperatures at the set points were determined by a temperature calibration using an external thermocouple. These are shown in Table S1 in the supporting information. For simplicity, the data sets will be referred to by their set point temperatures.

Data were also collected in a similar fashion for a sample sealed under ambient air. These were treated via identical procedures to the ones described above. The air sample was found to decompose via oxidation at elevated temperature, which compromised the analyses. The experimental details and primary findings from this sample are reported in the supporting information.

### 2.2. Theoretical computations

Calculations of the static structure factors and theoretical ADPs of diamond were conducted by DFT calculations using the Perdew–Burke–Ernzerhof (PBE) functional (Perdew *et al.*, 1996 ▸). Experimental lattice parameters extracted from preliminary Rietveld models were used for the calculations. Theoretical structure factors were calculated based on the full EDs from DFT calculations using a full-potential linear augmented plane wave plus local orbitals method in the *Wien2k* code (Blaha *et al.*, 2020 ▸). The full ED calculations were conducted with a dense 46×46×46 *k* mesh, a plane-wave cutoff parameter *R*_MT_ *K*_max_ of 10, an energy convergence criterion of 10^−6^ eV and spherical harmonics up to *l*_max_ = 10 for the expansion of ED inside the atomic spheres. Structure factors up to a resolution of sin(θ)/λ < 1.75 Å^–1^ were obtained by Fourier transformation of the calculated total EDs. The harmonic phonon calculations (HPC) were conducted by combining *VASP* (Blöchl, 1994 ▸; Kresse & Furthmüller, 1996 ▸) and *Phonopy* (Togo & Tanaka, 2015 ▸) using the finite displacement method (Parlinski *et al.*, 1997 ▸) with a default displacement amplitude of 0.01 Å. DFT calculations of atomic forces by *VASP* were conducted in supercells with 64 atoms (2×2×2 conventional cells) using a plane-wave energy cutoff of 800 eV, an energy convergence of 10^−8^ eV and an 11×11×11 Monkhorst–Pack *k* mesh. The isotropic ADPs were obtained using a dense 58×58×58 *q* mesh based on the HPCs as implemented in *Phonopy*.

### 2.3. Structure factor extraction

The observed structure factors *F*_obs_ were extracted from the PXRD data via the iterative WHCR procedure (Bindzus *et al.*, 2014 ▸). The Hansen–Coppens–Rietveld model was established in the *JANA2006* software (Petříček *et al.*, 2014 ▸) and included the following parameters: scale factor, lattice constant *a*, zero point shift, isotropic ADP, peak profile parameters (*U*, *V*, *W*, *X* and *Y*) for a left–right split pseudo-Voigt peak shape function (up to ten parameters in total), and multipole parameters including the expansion/contraction parameters (κ_v_ and 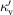 ) and population parameters for the symmetry-allowed octo- and hexadecapoles, *P*_32−_, *P*_40_ and *P*_44+_. The first two are sometimes denoted *O*2− and *H*0 in the literature. The last is restricted to *P*_40_ times a scaling factor in the space-group symmetry of diamond. The multipole model was set up in a right-handed coordinate system, which causes *P*_32−_ to have the opposite sign to other reports using a left-handed system. Modelled Bragg peaks were cut at 25 times their FWHM and the background was described by linear interpolation between 22 and 27 manually selected background points. The multipoles were set up with radial functions based on *sp* ^3^ hybridization of the carbon atoms, which were generated by Slater-type orbitals (STOs) of the Cv atom in the Su–Coppens–Macchi (SCM) scattering bank (Macchi & Coppens, 2001 ▸). The value of the single ζ exponents was set at 3.156 Bohr^−1^ and the electronic configuration to 1*s* ^2^2*s* ^1^2*p* ^3^. Core and valence population coefficients were not refined. The lower and upper limits of the angular range were set to 11° and 110°, respectively, in 2θ, corresponding to sin(θ)/λ = 1.67 Å^−1^ (*q* = 21.0 Å^−1^). The observed structure factors from refinement of this model were then used in a Wilson plot according to equation (2). The extracted ADP from the Wilson plot was then used for the consecutive iteration of the multipole Rietveld model, for which all parameters except the ADP were refined. Iterations were repeated until the ADP had converged on the sixth decimal, which required between two and five iterations for the ten different data sets. Models that included anharmonic thermal motion via the Gram–Charlier formalism (Zucker & Schulz, 1982 ▸; Kuhs, 1992 ▸; Trueblood *et al.*, 1996 ▸) with parameters of up to the fourth order were also tested with and without multipole parameters. In anharmonic models with multipole parameters, the third- and fourth-order Gram–Charlier parameters refined to virtually zero and the agreement factors did not improve. In the anharmonic models without multipole parameters, the agreement factors were poorer than those obtained from the harmonic models with multipole parameters. Harmonic anisotropic thermal motion was not tested as the anisotropic coefficients are forbidden by the site symmetry of carbon in the diamond space group.

### 2.4. Multipole modelling and chemical bonding analysis

The extracted structure factors from the WHCR procedure were used alongside the refined unit-cell parameters and ADPs to model the aspherical ED in *XD2016* (Volkov *et al.*, 2016 ▸). The structure factors from *JANA2006* are already corrected for anomalous dispersion and were therefore not further corrected in *XD2016*. The refinement routine consists of first refining κ and then fixing it, followed by including the multipole function incrementally in *l*, before finally including both *κ*_v_ and 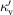 . All reported models used the estimated ADPs from Wilson plot fitting and convergence was set at Δ*x*/*s_x_* < 10^−10^ ∀ *x*, where *x* represents the model parameters and *s_x_* its associated uncertainty. Refinement of the ADP and the inclusion of anharmonic thermal motion parameters before refining the aspherical density were tested, but both models showed worse agreement with theoretical ADPs. From the refined models, the single C—C BCP is located within a cluster of 4×4×4 unit cells. The density and Laplacian were evaluated at the BCP. For comparison, the structure factors from the theoretical computations were also subjected to a similar analysis in *XD2016* without thermal motion.

## 3. Results and discussion

**Figure 1:**
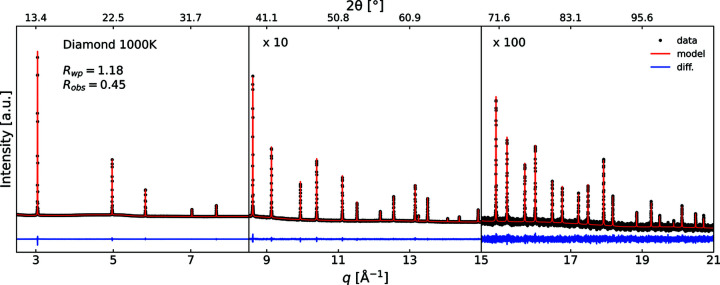
The multipole Rietveld model from the final iteration of the WHCR procedure for diamond at 1000 K. Agreement factors and refined parameters for all measured temperatures are shown in Tables S1–S3.

**Figure 2:**
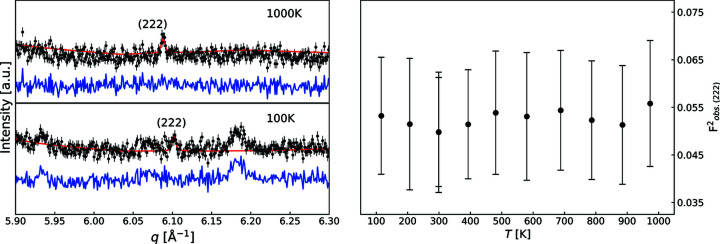
(Left) WHCR models for the (222) reflection from diamond at 100 K and 1000 K. (Right) Observed squared structure factors for the (222) reflection corrected for thermal motion.

Extraction of the observed structure factors was carried out using the WHCR procedure. The powder diffractogram and final WHCR model for the 1000 K data are shown in Fig. 1 ▸. The visual conformity and good agreement factors show that the model accurately describes the data. Models for all the other temperatures are of similar quality. Weak reflections from an impurity phase were observed in the data up to a temperature of 800 K; see Fig. S1 for the most significant peaks. The impurity reflections disappear at higher temperatures and do not re-emerge upon cooling, which strongly suggests that they stem from a powder stuck to the outside of the sample capillary. The impurity phase could not be successfully identified. There was no significant peak overlap between the impurity and diamond phases, so the majority of impurity peaks could be masked from the WHCR model. In the left-hand panel of Fig. 2 ▸ the (222) reflection at 100 K and 1000 K is shown, and the observed structure factors at all measured temperatures are given in the right-hand panel. The ‘forbidden’ (222) reflection is clearly visible in the 1000 K data but less pronounced in the 100 K data. The peak at *q* = 6.18 Å^−1^ in the 100 K data is also attributed to the impurity phase as it disappears upon heating. Even though the uncertainties on the observed (222) structure factors are relatively large due to the low signal-to-noise ratio, the data quality is sufficient to observe effects from chemical bonding.

**Figure 3:**
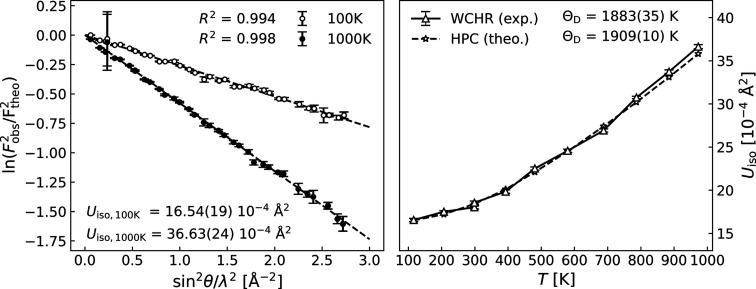
(Left) Final Wilson plots for the 100 K (open circles) and 1000 K (closed circles) WHCR models after two and three iterations, respectively. (Right) Theoretically calculated ADPs from HPC and experimentally extracted ADPs. The Debye model fits can be seen in the supporting information.

Wilson plots for the observed structure factors at 100 K and 1000 K are shown in the left-hand panel of Fig. 3 ▸. In accordance with equation (2), the ratios between observed and static structure factors fall extremely close to straight lines, as evident from the coefficients of determination, *R* ^2^. The linear fits to the Wilson plots for all the other temperatures are of similar quality. This demonstrates that by refining the multipole parameters it is possible to find sets of observed structure factors which have excellent agreement with the observed intensity and are consistent with isotropic and harmonic thermal motion. In the right-hand panel of Fig. 3 ▸ the experimental ADPs for all measured temperatures are shown alongside the theoretical values from HPC. Excellent agreement between the two is observed, which suggests that the experimental values are accurate. These observations lead to the conclusion that the thermal motion in diamond is predominantly isotropic and harmonic even at 1000 K, which is also corroborated by the following: the intensities of the forbidden reflections of the diamond-type structures of silicon (Si) and germanium (Ge) show a distinct ‘crossover’ temperature where the intensity reaches a minimum. This is caused by the competing effects of aspherical deformation and anharmonic thermal motion. At elevated temperatures, the effect of anharmonic thermal motion dominates, causing the crossover temperature for the (442) reflection in Si to be approximately 525 K (Trucano & Batterman, 1972 ▸). As shown in Fig. 2 ▸ (left panel), such a crossover temperature is not observed for the (222) reflection of diamond, which also confirms that effects from anharmonicity are negligible in the explored temperature range.

**Figure 4:**
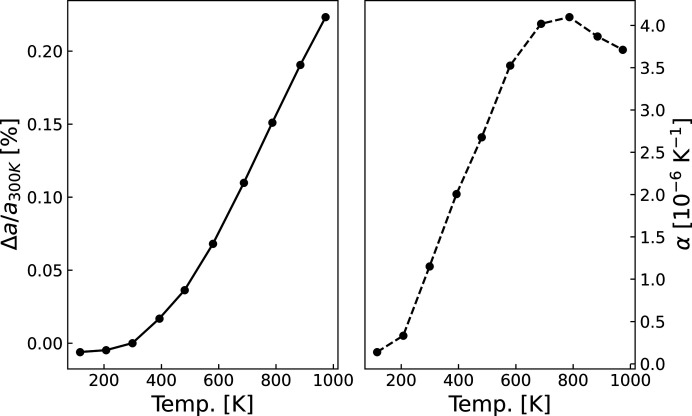
(Left) The increase in the refined lattice parameters from the final WHCR models at the measured temperatures. Δ*a* is the difference from the lattice parameter at 300 K. The latter was calculated as the average of the two refined lattice parameters at 300 K (‘300K’ and ‘300 K-after’ data sets). The refined values are shown in Table S2. (Right) The linear thermal expansion coefficient α at the measured temperatures. This was computed as the gradient of the refined lattice parameters normalized to the 300 K value [computation done using the numpy.gradient() function from the *numpy* Python library; see https://numpy.org/doc/stable/reference/generated/numpy.gradient.html for documentation].

Nevertheless, these effects cannot be completely discarded. Anharmonicity stems from non-harmonic potentials in the energy landscape between neighbouring atoms, which causes their equilibrium interatomic distances to change with the energy of vibrational states. In crystallography, the non-harmonic potentials are ubiquitously described by a Gram–Charlier expansion of the harmonic potential (Zucker & Schulz, 1982 ▸; Kuhs, 1992 ▸; Trueblood *et al.*, 1996 ▸). Lattice thermal expansion, which is observed as the increase in refined lattice parameters depicted in Fig. 4 ▸ (left panel), can only be explained by anharmonicity since the equilibrium interatomic distances do not change in a harmonic potential. As such, the effects of anharmonicity were tested by preliminary Rietveld models via inclusion of third- and fourth-order Gram–Charlier parameters, but these did not improve the models. This may be consolidated, however, by the minute thermal expansion from 100 K to 1000 K, which is only slightly above 0.8 pm (corresponding to a 0.23% increase). In conclusion, the effects from anharmonicity on the observed ED are negligible within the precision of the experiments.

The refined lattice parameter values at 300 K, the expansion coefficient at 300 K (α = 1.15 × 10^−6^ K^−1^) and its significant increase from 100 K to 700 K are all in excellent agreement with previously reported results (Reeber & Wang, 1996 ▸; Jacobson & Stoupin, 2019 ▸). The small, albeit statistically significant, uncertainty between the two refined lattice parameters at 300 K [*a* = 3.567141 (4) Å and *a* = 3.567175 (3) Å from the ‘300K’ and ‘300 K-after’ data sets, respectively] is within the uncertainty of the calibrated wavelength.

The Debye temperatures Θ_D_ of diamond reported in the left-hand panel of Fig. 3 ▸ were determined via the following formulation of the Debye model, which applies for homo­atomic cubic structures (Willis & Pryor, 1975 ▸; Bentien *et al.*, 2005 ▸; Fischer *et al.*, 2018 ▸): 

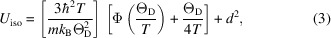

where

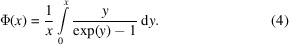

Here, *m* is the atomic mass, *k*_B_ the Boltzmann constant, ℏ the reduced Planck constant, *T* the temperature and *d* ^2^ an empirical term for describing temperature-independent disorder. The fitted Debye temperatures are 1883 (35) K and 1909 (10) K for the ADPs extracted from the WHCR procedure and those from the HPC, respectively. The good agreement once again corroborates the predominantly isotropic and harmonic thermal motion in diamond. The refined values of the disorder parameters *d* ^2^ for the experimental ADPs were virtually zero, suggesting a miniscule degree of disorder.

**Figure 5:**
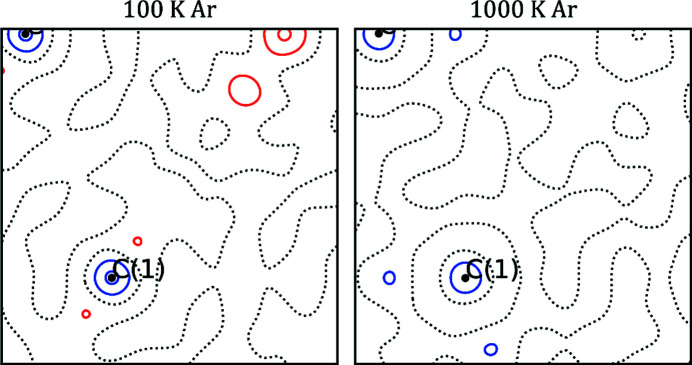
Residual density maps in the plane of two C—C bonds. The contour lines are drawn at intervals of 0.10 e Å^−3^. Black dotted lines represent the zero contour, and blue and red represent positive and negative values, respectively.

The extracted structure factors were used to model the ED in the *XD2016* software and the agreement factors plus refined parameters from all models are given in Table 1 ▸. Residual density maps in the plane of two C—C bonds for the models against the 100 K and 1000 K data are shown in Fig. 5 ▸. The residual densities for both samples are extremely flat and most residuals are located in the core region. This is to be expected given the negligence of core deformation in the models (Bindzus *et al.*, 2014 ▸). Fractal dimension plots (Meindl & Henn, 2008 ▸) for the 100 K and 1000 K data are shown in the supporting information. These show extremely narrow and parabola-like distributions of residuals, corresponding to small and normally distributed errors.

**Figure 6:**
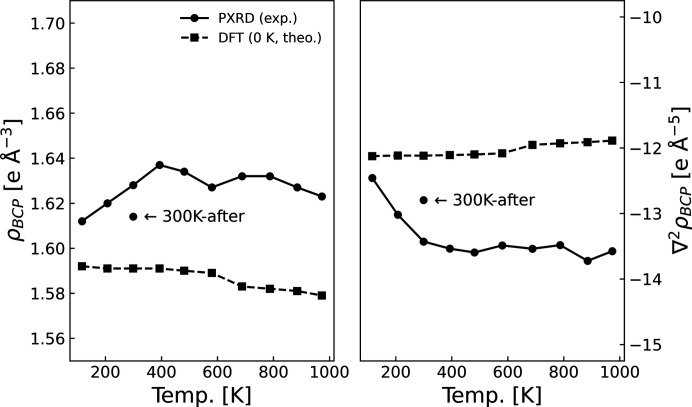
(Left) The BCP density, ρ_BCP_, and (right) the Laplacian at the BCP, ∇^2^ρ_BCP_, as a function of temperature using the experimentally obtained (circles) and theoretically calculated (squared) structure factors. The latter were calculated at 0 K but with lattice parameters extracted from the experimental data.

The evaluated ED and Laplacian in the BCP (ρ_BCP_ and ∇^2^ρ_BCP_, respectively) are plotted against temperature in Fig. 6 ▸. The Laplacian ∇^2^ρ_BCP_ represents the concentration or depletion of electrons at the BCP relative to the local environment, and its sign can therefore be used to distinguish different types of bond. Covalent bonds are expected to show an accumulation of electrons into the bond, and consequently ∇^2^ρ_BCP_ will be negative as the ED decreases rapidly upon moving perpendicular to the bond. Ionic bonds, on the other hand, are expected to deplete the ED at the BCP, giving a positive sign for the Laplacian. It is possible to calculate a wealth of other properties within the QTAIM framework (Bader, 1994 ▸; Gatti, 2005 ▸), but the scope of the current study is limited to the BCP density and Laplacian. The relatively large negative value of ∇^2^ρ_BCP_ corroborates that the C—C interaction in diamond is covalent, in line with expectations from fundamental chemistry. The value of ρ_BCP_ ranges from 1.61 to 1.64, which is in excellent agreement with previous studies (Svendsen *et al.*, 2010 ▸; Bindzus *et al.*, 2014 ▸; Deguchi & Nishibori, 2018 ▸; Svane *et al.*, 2021 ▸).

To confirm that the topological parameters should remain constant when the thermal motion is properly decoupled, ρ_BCP_ and ∇^2^ρ_BCP_ were also determined from models against the theoretically calculated structure factors. These were calculated without thermal motion but in geometries that take the thermal expansion into account. Small differences in the calculated structure factors for the 100 K and 1000 K lattice parameters were observed but, since the thermal expansion is minute, the corresponding topological parameters are nearly identical at all temperatures. In conclusion, the chemical bonds do not change as a consequence of the thermal expansion in the theoretical case. The small decreasing trends in ρ_BCP_ and increasing trend in ∇^2^ρ_BCP_ can be explained by the small volumetric increase in the unit cell, resulting in a lower average ED. Aside from the small increase in ρ_BCP_ and decrease in ∇^2^ρ_BCP_ from 100 K to 300 K, the experimentally obtained topological parameters are also stable with temperature. This confirms the validity of the convolution approximation in the case of diamond in the explored temperature range.

Concerning the small trends at low temperature, the variation between the ‘100K’ and ‘300K’ data is of the same order of magnitude as that between the ‘300K’ and ‘300K-after’ data sets. This suggests that the variation is a numerical consequence of the model and not a physical consequence of the thermal motion, and this is also corroborated by the stability of ρ_BCP_ and ∇^2^ρ_BCP_ at high temperature. If the convolution approximation was violated, one would expect the discrepancy to be exacerbated by rigorous thermal motion at high temperature. An offset between experimental and theoretical values was also observed by Bindzus *et al.* (2014 ▸) and is attributed to a shortcoming of the PBE functional for accurately calculating the ED of diamond.

The value of ρ_BCP_ for the ‘300K’ and ‘300K-after’ data changes from 1.628 to 1.614 Å^−3^, respectively, and ∇^2^ρ_BCP_ from −13.4 to −12.8 Å^−5^, respectively. The uncertainties on ∇^2^ρ_BCP_ are unknown but the Laplacian is a rapidly changing function known to be sensitive to even minor changes in the model, especially BCP positions (Kamin’ski *et al.*, 2014 ▸; Fournier *et al.*, 2018 ▸; Shi *et al.*, 2019 ▸). As such, it is probably also sensitive to minor changes in experimental conditions, such as fluctuations in the incident beam. On the other hand, the ‘300K-after’ data were collected after rapid cooling from 1000 K, which might have an effect on the structure. It is not possible to conclude whether this disagreement stems from experimental and/or model errors, or if it is a structural effect. However, it should also be noted that the values of refined ADPs from the final iteration of the different 300K models are not identical. This suggests a shortcoming in the precision and robustness of the WHCR models. The ADPs for the two models are 18.02 (19) × 10^−4^ Å^2^ and 18.55 (19) × 10^−4^ Å^2^. These values should be compared with the 18.19 × 10^−4^ Å^2^ as found by Bindzus *et al.* (2014 ▸) at the same temperature with a similar procedure. One of the primary sources of error in the extraction of structure factors from PXRD experiments is treatment of the background. This significantly alters the intensity of weak reflections with low signal-to-noise ratios, such as the (222) reflection and those at high order. To assess the general robustness of the WHCR procedure, the ‘300K-after’ data set was modelled five times with different selections of background points. As reported in Table 2 ▸, the ADPs span a range from 17.89 × 10^−4^ Å^2^ to 18.59 × 10^−4^ Å^2^, which is a rather large variation considering that the models are refined against the same data set. The variation is primarily a consequence of the inaccuracy of the extracted structure factors at high order, upon which the slope of the linear Wilson plot fits is very dependent. Fig. S4 shows a comparison of extracted structure factors between the ‘300K-after’ and the four models with differently selected background points (labelled ‘300K-after II’ to ‘300K-after V’). A significant variation of up to 5% in the structure factors is observed for the weak (222) reflection and the high-order reflections. This points to an underlying inaccuracy in the extraction of structure factors from PXRD using the WHCR procedure.

To assess the corresponding effects on modelling in *XD2016*, the multipole and topological parameters for the five different models of the ‘300K-after’ data are shown in Table 2 ▸. Fractal dimension plots for the models are shown in Fig. S5. Fortunately, the variation is much less pronounced for the multipole and topological parameters than for the ADPs, as evident from the stability of parameters (Table 2 ▸) and the similarity in fractal dimension plots (Fig. S5). The reason is that the ADP predominantly affects high-order structure factors [sin(θ)/λ > 0.5 Å^−1^], whereas the information from the valence ED is concentrated in the low-order structure factors [sin(θ)/λ < 0.5 Å^−1^] (Bindzus *et al.*, 2014 ▸). The two parameters ρ_BCP_ and ∇^2^ρ_BCP_ only range from 1.612 Å^−3^ to 1.616 Å^−3^ and −12.847 Å^−3^ to −12.635 Å^−3^, respectively. In conclusion, the ambiguity in the selection of background points cannot explain the differences between the ‘300K’ and ‘300K-after’ models in Fig. 6 ▸, and the reason remains unclear.

## 4. Conclusions

Structure factors extracted from PXRD data via an iterative Wilson–Hansen–Coppens–Rietveld (WHCR) procedure were used to demonstrate that the thermal motion in diamond is predominantly harmonic and isotropic in the temperature range from 100 K to 1000 K. HC modelling against the extracted structure factors led to accurate fits with good agreement factors and extremely low residual densities.

Subsequent topological analysis of the ED has demonstrated a relative constancy in the density and Laplacian at the BCP as a function of temperature. This leads to the conclusion that the ED is unperturbed by the increased temperature, which demonstrates that the thermal motion can be completely deconvoluted from the static ED, thus confirming the convolution approximation for diamond in the measured temperature range.

The Debye temperature of diamond was determined experimentally to be Θ_D_ = 1883 (35) K and theoretically to be Θ_D_ = 1909 (10) K. The robustness of the WHCR procedure for structure factor extraction from PXRD is compromised by small differences in treatment of the background signal, which mainly affects the precision in refined ADPs. However, the imprecision does not affect the subsequent HC models significantly.

The collected diffractograms for diamond and LaB_6_ are available in the supporting information, together with the extracted structure factors using the WHCR procedure. The calculated structure factors from DFT are also supplied.

## 5. Related literature

For further literature related to the supporting information, see Bansal *et al.* (1972 ▸), Barrer (1936 ▸) and Moelle *et al.* (1997 ▸).

## Supplementary Material

Additional tables and figures. DOI: 10.1107/S2053273322010154/pl5020sup1.pdf

Diffractograms and extracted structure factors. DOI: 10.1107/S2053273322010154/pl5020sup2.zip

## Figures and Tables

**Table 1:** Relevant refined parameters for all measured temperatures The ADPs are those extracted from the WHCR procedure, while the agreement factors, multipole and topological parameters are the ones from refinement in *XD2016*.

Sample name	*R*(*F* ^2^) (%)	*U*_iso_ (× 10^−4^ Å^2^)	Scale factor	κ	*κ*′	*P*_32−_ †	*P*_40_	ρ_BCP_ (e Å^−3^)	∇^2^ρ_BCP_ (e Å^−5^)
100 K	0.84	16.54 (19)	0.9972 (8)	0.965 (4)	0.859 (15)	−0.38 (1)	−0.12 (2)	1.612 (0)	−12.459 (1)
200 K	0.59	17.50 (12)	0.9974 (6)	0.958 (3)	0.844 (12)	−0.37 (1)	−0.17 (2)	1.620 (0)	−13.019 (1)
300 K	0.72	18.02 (19)	0.9975 (7)	0.956 (4)	0.857 (14)	−0.37 (1)	−0.17 (2)	1.628 (0)	−13.429 (1)
300 K-after	0.69	18.55 (19)	0.9974 (7)	0.961 (3)	0.858 (12)	−0.37 (1)	−0.14 (2)	1.614 (0)	−12.797 (1)
400 K	0.70	19.83 (20)	0.9970 (8)	0.960 (4)	0.877 (14)	−0.37 (1)	−0.14 (2)	1.637 (0)	−13.538 (2)
500 K	0.58	22.51 (19)	0.9970 (6)	0.955 (3)	0.852 (11)	−0.37 (1)	−0.18 (2)	1.634 (0)	−13.596 (1)
600 K	0.61	24.57 (20)	0.9972 (6)	0.954 (3)	0.845 (11)	−0.36 (1)	−0.19 (2)	1.627 (0)	−13.489 (1)
700 K	0.47	26.91 (18)	0.9973 (6)	0.953 (3)	0.857 (10)	−0.39 (1)	−0.15 (2)	1.632 (0)	−13.539 (1)
800 K	0.55	30.74 (15)	0.9973 (6)	0.957 (3)	0.853 (10)	−0.38 (1)	−0.16 (2)	1.632 (0)	−13.484 (1)
900 K	0.60	33.73 (22)	0.9977 (6)	0.943 (3)	0.843 (9)	−0.39 (1)	−0.17 (2)	1.627 (0)	−13.724 (1)
1000 K	0.54	36.63 (24)	0.9980 (6)	0.945 (3)	0.849 (11)	−0.38 (1)	−0.16 (2)	1.623 (0)	−13.576 (1)

† A right-handed coordinate system was used for the multipole model, which accounts for the negative sign compared with other studies, such as Bindzus *et al.* (2014 ▸) and Svane *et al.* (2021 ▸).

**Table 2:** Relevant refined parameters for the five different models of the ‘300K-after’ data set The lattice constants and ADPs are those extracted from the Rietveld–Wilson procedure, while the agreement factor, multipole and topological parameters are the ones found from refinement in *XD2016*.

	300K-after	300K-after II	300K-after III	300K-after IV	300K-after V
Lattice constant *a* (Å)	3.567175 (3)	3.567175 (3)	3.567174 (3)	3.567175 (3)	3.567175 (3)
*U*_iso_ (× 10^−4^ Å^2^)	18.55 (19)	17.89 (17)	18.25 (19)	18.23 (20)	18.59 (21)
*R*_wp_ (%)	1.21	1.21	1.19	1.19	1.19
*R*(*F* ^2^) (%)	0.69	0.71	0.71	0.73	0.72
κ_v_	0.961 (3)	0.964 (3)	0.964 (3)	0.962 (3)	0.959 (3)
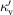	0.858 (12)	0.865 (13)	0.858 (13)	0.858 (13)	0.858 (13)
*P*_32−_	−0.37 (1)	−0.37 (1)	−0.37 (1)	−0.37 (1)	−0.36 (1)
*P*_40_	−0.14 (2)	−0.13 (2)	−0.14 (2)	−0.15 (2)	−0.15 (2)
∇^2^ρ_BCP_ (e Å^−5^)	−12.797 (1)	−12.748 (1)	−12.635 (1)	−12.847 (1)	−12.840 (1)
ρ_BCP_ (e Å^−3^)	1.614 (1)	1.616 (1)	1.612 (1)	1.617 (1)	1.613 (1)
