# Supplementary material for: Electron density and thermal motion of diamond at elevated temperatures
Source: Acta Crystallogr A Found Adv. 2023 Jan 1;79(Pt 1):41–50. doi: 10.1107/S2053273322010154 (PMC9813686; doi:10.1107/S2053273322010154)
Supplement: Supplementary file 1 [file a-79-00041-sup1.pdf]

Acta Cryst A FOUNDATIONS ADVANCES

Volume 79 (2023)

Supporting information for article:

Electron density and thermal motion of diamond at elevated temperatures

Jonas Beyer, Thomas Bjørn Egede Grønbech, Jiawei Zhang, Kenichi Kato and Bo Brummerstedt Iversen

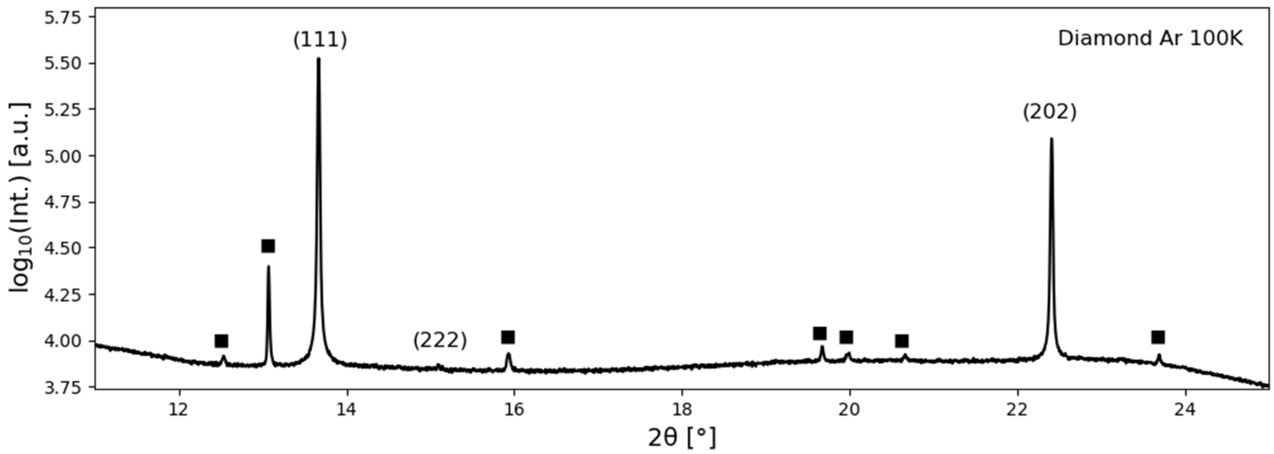

Figure S1. PXRD data from the diamond sample at 100 K. Filled squares denote the most significant reflections from impurity phase, which has not been identified.

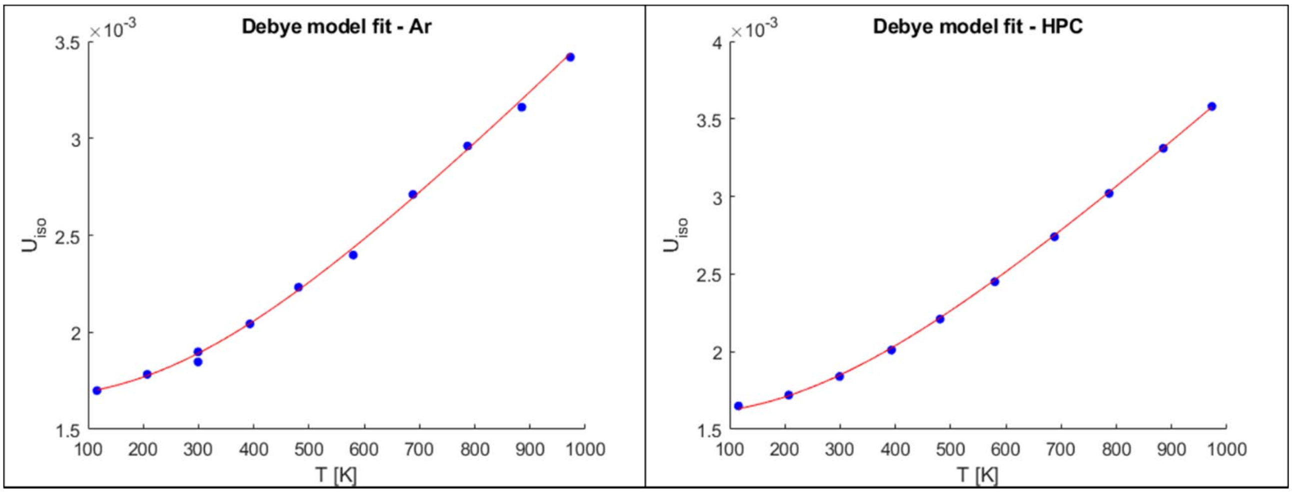

Figure S2. Debye model fits to the (left) ADPs extracted from the WHCR procedure and (right) values from the HPC. The Debye temperatures can be seen in Figure 3.

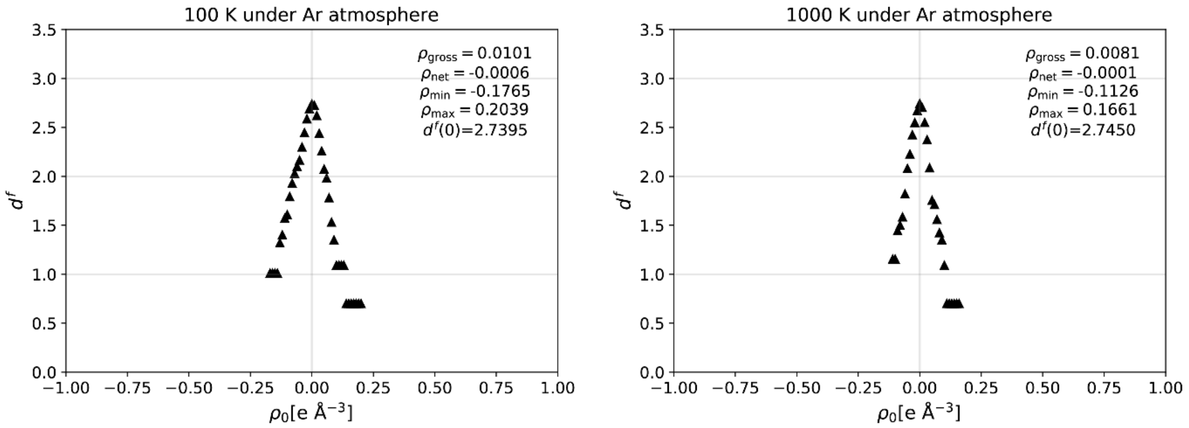

Figure S3. Fractal dimension plots for models against the 100K and 1000K data sets.

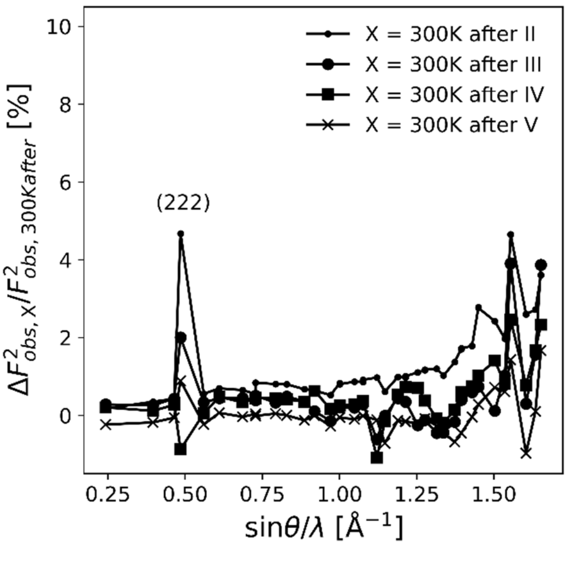

Figure S4.Comparison of extracted structure factors from the 300K-after data with four models using different background points.

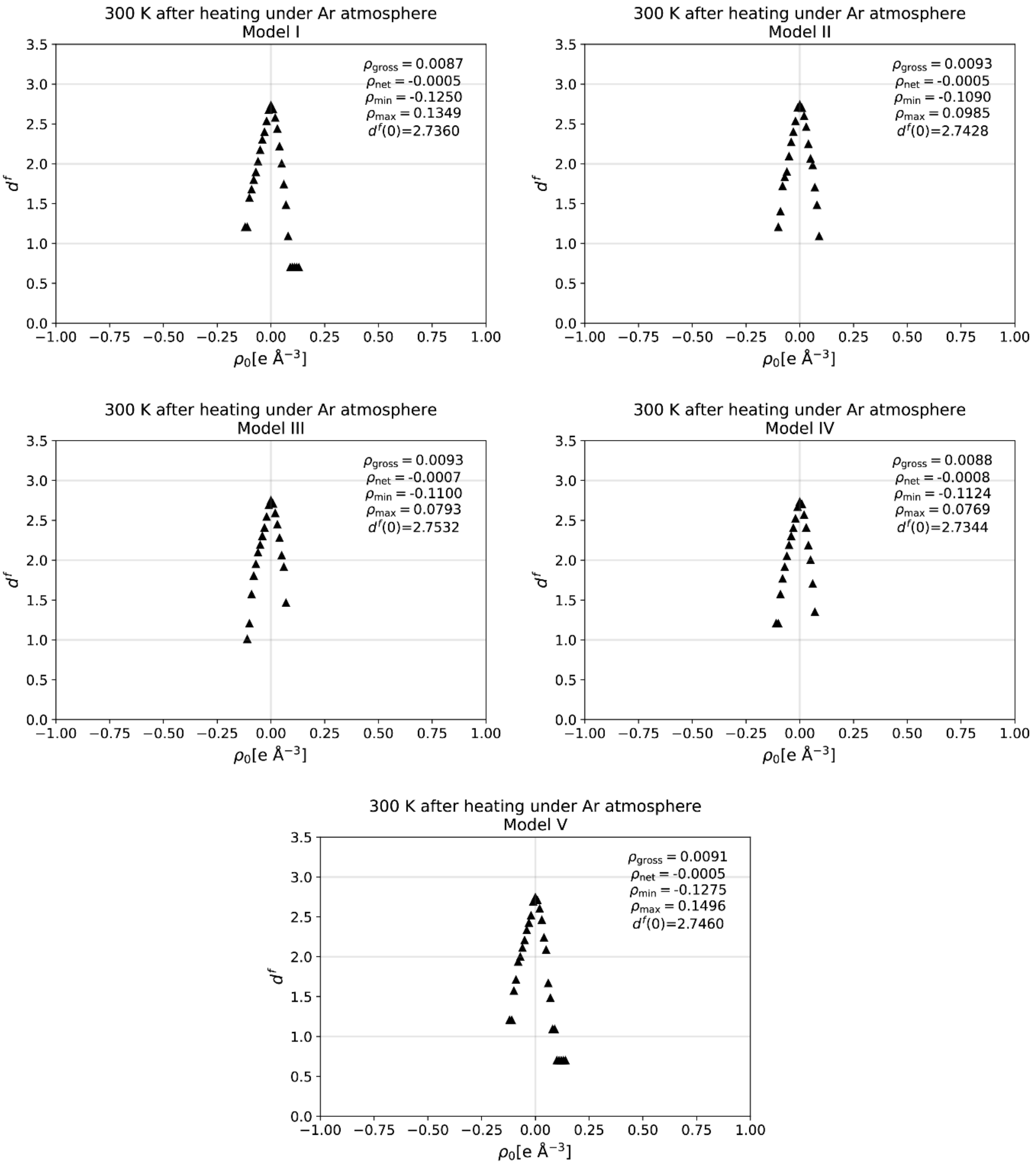

Figure S5. Fractal dimension plots for the five different models of the 300K-after data set using different background points.

Table S1. Relevant parameters for the reported experiments.

Sample name	Calib. Temp. [K]	R_{wp} [%]	R_{obs} [%]	JANA Scale	Zero point shift	Scale s_{F2}	U_{iso} [Å^2] from Wilson fitting	U_{iso} [Å^2]from HPC
100K	116	1.16	0.56	2.9237(14)	0	16.012	0.001654(19)	0.00165
200K	207	1.20	0.40	2.9312(14)	0	15.994	0.001750(12)	0.00172
300K	299	1.23	0.51	2.9404(14)	0	15.970	0.001802(19)	0.00184
300K-after	299	1.21	0.49	2.9470(14)	0	15.960	0.001855(19)	0.00184
400K	393	1.28	0.53	2.9397(15)	0	15.935	0.001983(20)	0.00201
500K	481	1.25	0.48	2.9366(14)	0	16.006	0.002251(19)	0.00221
600K	580	1.23	0.50	2.9380(14)	-0.223(6)	15.993	0.002457(20)	0.00245
700K	688	1.25	0.47	2.9314(15)	-0.224(6)	16.069	0.002691(18)	0.00274
800K	787	1.19	0.41	2.9149(15)	-0.105(6)	16.066	0.003074(15)	0.00302
900K	885	1.19	0.57	2.9320(14)	-0.124(6)	15.980	0.003373(22)	0.00331
1000K	973	1.18	0.45	2.9435(14)	-0.185(6)	16.050	0.003663(24)	0.00358

Table S2. Lattice constant and HC multipole parameters refined in *JANA 2006* from the final iteration of the WHCR procedure.

Sample name	Lattice const. *a* [Å]	κ_v	κ_v'	P_{32-}^*	P_{40}^*
100K	3.566940(3)	0.965(3)	0.864(9)	-0.366(7)	-0.123(12)
200K	3.566985(3)	0.957(3)	0.845(9)	-0.359(8)	-0.171(15)
300K	3.567141(4)	0.956(3)	0.863(9)	-0.353(7)	-0.166(14)
300K-after	3.567175(3)	0.959(3)	0.864(9)	-0.367(7)	-0.142(13)
400K	3.567758(4)	0.960(3)	0.885(9)	-0.371(7)	-0.134(12)
500K	3.568451(4)	0.954(3)	0.854(9)	-0.360(8)	-0.181(14)
600K	3.569583(6)	0.955(3)	0.858(9)	-0.354(7)	-0.185(14)
700K	3.571075(6)	0.952(3)	0.861(9)	-0.376(7)	-0.157(14)
800K	3.572541(6)	0.956(3)	0.860(9)	-0.371(7)	-0.169(14)
900K	3.573954(6)	0.943(3)	0.850(8)	-0.382(7)	-0.176(14)
1000K	3.575119(6)	0.945(3)	0.855(8)	-0.377(7)	-0.167(14)

Table S3. Peak profile parameters for the left-right split Thompson-Cox-Hastings pseudo-Voigt peak profile function from the final iteration of the Rietveld-Wilson procedure. *Parameter was fixed to the stated value.

Sample	Left					right				
name	U	V	W	LX	LY	U	V	W	LX	LY
100K	0*	0.89(5)	0.463(8)	0.372(9)	4.014(5)	0*	-0.25(5)	0.711(8)	0.338(9)	3.48(5)
200K	0*	1.06(5)	0.418(8)	0.416(9)	3.870(5)	0*	-0.32(4)	0.730(8)	0.348(9)	3.41(5)
300K	0*	1.11(5)	0.390(8)	0.428(9)	3.877(5)	0*	-0.33(4)	0.744(8)	0.347(9)	3.40(5)
300K-after	0*	1.18(5)	0.391(8)	0.441(9)	3.203(5)	0*	-0.08(4)	0.702(8)	0.380(9)	2.92(5)
400K	0*	1.18(6)	0.349(8)	0.444(10)	3.915(5)	0*	-0.54(5)	0.789(8)	0.32(1)	3.62(5)
500K	0*	1.14(6)	0.312(8)	0.464(9)	3.735(5)	0*	-0.57(5)	0.811(8)	0.317(9)	3.53(5)
600K	0*	0.83(6)	0.576(12)	0.376(10)	3.896(5)	0*	-0.11(5)	0.53(1)	0.408(9)	3.34(5)
700K	0*	0.88(7)	0.554(13)	0.380(10)	3.886(5)	0*	0.00(0)	0.55(1)	0.39(1)	3.40(5)
800K	0*	0.87(6)	0.491(11)	0.384(10)	3.718(5)	0*	-0.04(5)	0.60(1)	0.39(1)	3.72(5)
900K	0*	1.04(4)	0.481(9)	0.386(5)	3.573*	0*	-0.02(3)	0.595(8)	0.396(5)	3.186*
1000K	0*	1.08(4)	0.537(9)	0.341(5)	3.573*	0*	0.05(3)	0.670(8)	0.377(5)	3.186*

Diamond sample under air

Experimental: A similar analysis to the one in the manuscript was carried out on a diamond sample sealed under ambient air instead of Argon. This lead to a slight decomposition during the multi-temperature PXRD experiment, causing an uncertainty in the topological parameters. This diamond powder was purchased from Nilaco (average particle size ~ 6 μm) and packed in one glass and one quartz capillary with inner diameters of 0.3 mm, which were used for low (≤ 300 K) and high (≥ 300 K) temperature measurements, respectively. PXRD were collected on the same instrument with an incident energy of 27.546(6) keV (λ = 0.45015(1) Å) as calibrated via le Bail model refinement against NIST LaB_6 standard reference material data set. The detector energy threshold was set to 13.6 keV. The spatial beam dimensions were 3.0 mm × 0.5 mm (horizontal × vertical) and 1.0 mm × 0.5 mm for the low and high temperature measurements, respectively. The temperature was set at points in a range from 100 K to 1000 K in 100 K steps and data were collected at 300 K with both the low and high temperature setups (referred to as '300K-LT' and '300K-HT', respectively). A heating rate of 60 K min^{-1} and 2 min waiting period were employed. The calibrated temperatures for the set points using an external thermocouple are given in Table S5. The employed WHCR procedure is identical to the one described in the Experimental section for the sample under Ar. The model range included data up to 101° 2θ corresponding to $\sin(\theta)/\lambda$ = 1.71 Å^{-1} (q = 21.5 Å^{-1}). Thermogravimetric analysis and differential scanning calometry (TGA-DSC) of the Sigma-Aldrich diamond powder (particle size ≤ 1 μm) were performed on a Netzsch STA 449 C Jupiter® apparatus using corundum (Al_2O_3) crucibles with lids. The instrument was calibrated against the melting points of In, Sn, Bi, Zn, Ag, and Au before use. Two experiments with different flow gasses were conducted: One with air (40 mL min^{-1}) and one with Ar (50 mL min^{-1}). The amount of diamond powder for the two experiments was 11.303(1) and 22.525(1) mg, respectively. The samples were heated from 30 °C to 800 °C and back to 30 °C with a heating/cooling rate of

5 °C min^{-1}. Empty crucibles were measured under similar conditions prior to the experiments for subtraction of instrumental contributions.

Results: The obsverved increase in ρ_{BCP} and decrease in $\nabla^2\rho_{BCP}$ for the sample under air may be related to oxidation of diamond at elevated temperatures. The powder samples comprised of micron-sized crystallites with relatively large surface areas compared to bulk samples. The surface of diamond has been reported to undergo chemisorption of O_2 followed by an oxidation to CO_2 above 600 K (Barrer, 1936; Bansal *et al.*, 1972; Moelle *et al.*, 1997. To confirm this, TGA-DSC was performed on the powder with a particle size below 1 µm under air and Ar. The measured mass losses are showed in Figure 6. Almost the entire sample under air disappears during the experiment, with a mass loss onset at 800 K, whereas the sample is completely stable under Ar. Obviously, the sample under air does not undergo the same degree of oxidation in the PXRD experiment, due to the closed environment inside the quartz capillary. However, chemisorption and oxidation must inevitably take place with the small amount of O_2 left inside causing the surface of the crystallites to change. The coincidence of the measured oxidation temperature with the fall-off in Laplacian therefore lead to the conclusion that the fall-off is a structural effect and not a matter of model error. This interpretation is further assisted by the agreement in $\nabla^2\rho_{BCP}$ between the samples in the temperature range 300 K to 700 K.

Figure S6 – (left) Final Rietveld model at the (222) reflection for the sample under air at 100 K and 1000 K and (right) the observed structure factors at all temperatures corrected for thermal motion.

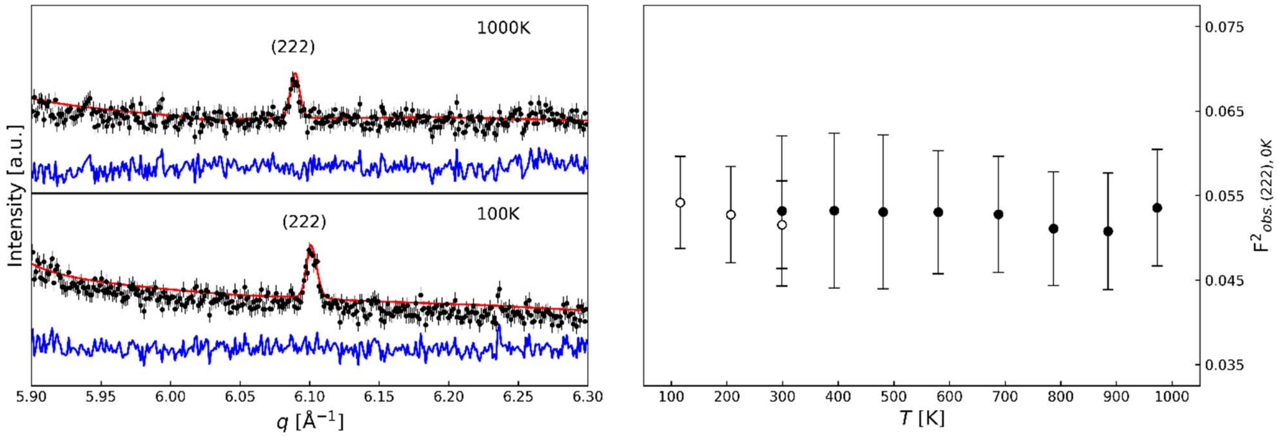

Figure S7. Residual density maps in the plane of two C-C bonds for the 100K and 1000K data for the sample under air. The contour lines are drawn in intervals of 0.10 e Å^{-3}, black dotted lines represent the zero contour, and blue and red represent positive and negative values, respectively.

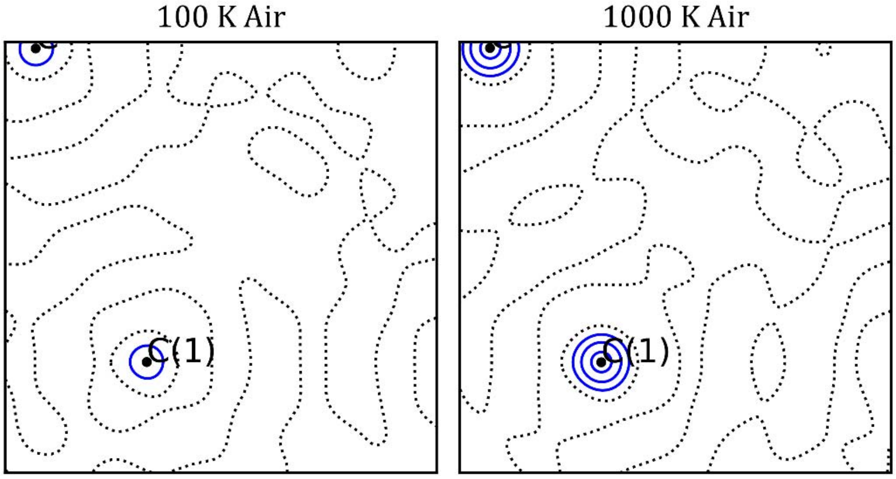

Figure S8. Fractal dimension plots for the samples under air at 100 K and 1000 K.

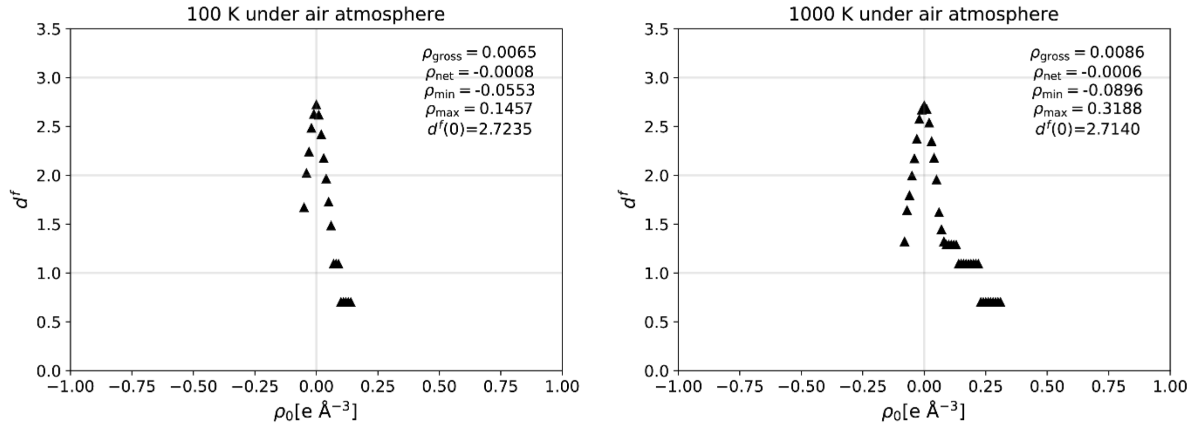

Figure S9. (left) Refined lattice parameters increase from the final WHCR models at the measured temperatures for the sample under air. The refined values are shown in Table S6. (right) The linear thermal expansion coefficient α at the measured temperatures.

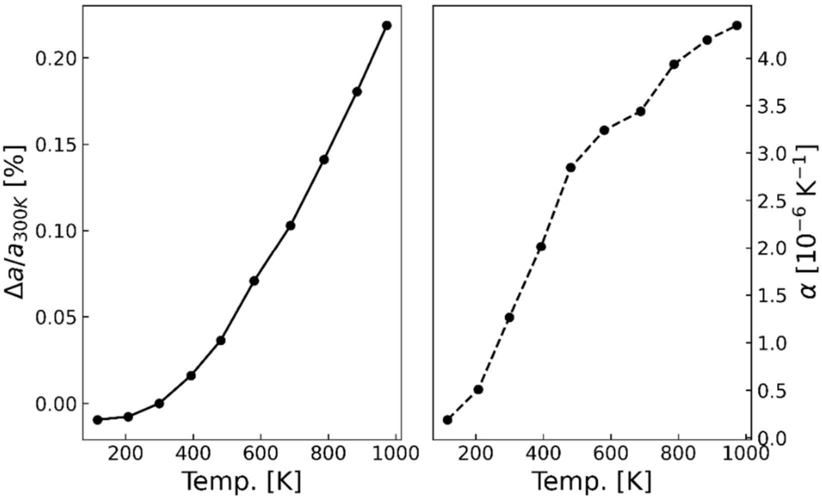

Figure S10. (left) BCP density, ρ_{BCP}, and (right) Laplacian at the BCP, $\nabla^2\rho_{BCP}$, as a function of temperature for the sample under air. The open circles denote the results from the '300K-HT' data set. The values are reported in Table S4.

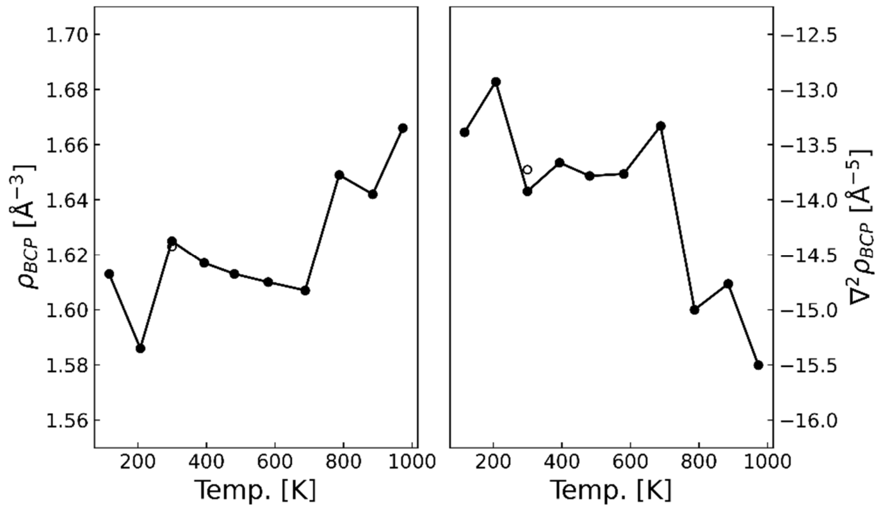

Figure S11. Relative mass loss from the TGA-DSC experiments on diamond powder (left) under a flow of air, and (right) under a flow of Ar.

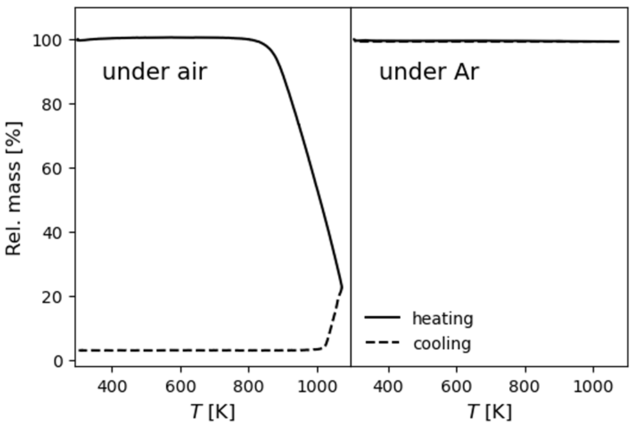

Table S4. Relevant refined parameters for the data collected on the sample under air. The ADPs are those extracted from the WHCR procedure, while the agreement factor, multipole, and topological parameters are the ones found from refinement in *XD2016*.

Sample name	$R(F^2)$ [%]	U_{iso} [10^{-4} Å^2]	Scale factor	κ	κ'	P_{32-}	P_{40}	ρ_{BCP} [e Å^{-3}]	$\nabla^2\rho_{BCP}$ [e Å^{-5}]
100K	0.56	16.99(13)	0.9985(5)	0.963(2)	0.952(9)	-0.30(1)	-0.12(1)	1.613(0)	-13.388(1)
200K	0.51	17.83(15)	0.9981(5)	0.947(2)	0.917(8)	-0.30(1)	-0.13(1)	1.586(0)	-12.930(1)
300K-LT	0.92	18.99(16)	1.0016(9)	0.960(3)	0.960(14)	-0.30(1)	-0.13(2)	1.625(0)	-13.924(2)
300K-HT	0.73	18.47(13)	0.9989(7)	0.965(3)	0.963(12)	-0.30(1)	-0.13(1)	1.623(0)	-13.727(2)
400K	0.65	20.43(16)	0.9988(6)	0.963(2)	0.953(10)	-0.30(1)	-0.14(1)	1.617(0)	-13.664(1)
500K	0.63	22.32(10)	0.9992(7)	0.955(2)	0.945(11)	-0.30(1)	-0.14(1)	1.613(0)	-13.784(1)
600K	0.75	23.98(20)	0.9986(8)	0.953(3)	0.952(13)	-0.30(1)	-0.14(1)	1.610(0)	-13.766(2)
700K	0.82	27.10(20)	0.9988(8)	0.963(3)	0.966(13)	-0.30(1)	-0.12(1)	1.607(0)	-13.331(2)
800K	0.86	29.60(10)	0.9987(3)	0.951(3)	0.942(13)	-0.32(1)	-0.16(1)	1.649(0)	-15.000(2)
900K	0.69	31.60(21)	0.9990(7)	0.947(3)	0.916(10)	-0.33(1)	-0.17(1)	1.642(0)	-14.766(1)
1000K	0.75	34.18(19)	0.9990(8)	0.949(3)	0.921(11)	-0.34(1)	-0.18(1)	1.666(0)	-15.502(2)

Table S5. Relevant parameters for the reported experiments with the sample under air

Sample name	Calib. Temp. [K]	R_{wp} [%]	R_{obs} [%]	JANA Scale	Zero point shift	Scale s_{F2}	U_{iso} [Å^2] from Wilson fitting	U_{iso} [Å^2]from HPC
100K	116	1.81	0.38	5.3186(25)	0.524(6)	16.021	0.001699(13)	0.00165
200K	207	2.00	0.38	5.3478(26)	0.555(6)	16.033	0.001783(15)	0.00172
300K-LT	299	2.01	0.42	5.3271(26)	0.568(6)	16.022	0.001899(16)	0.00184
300K-HT	299	1.47	0.49	3.9704(20)	-0.081(6)	16.062	0.001847(13)	0.00184
400K	393	1.53	0.44	3.9709(20)	-0.031(6)	16.048	0.002043(16)	0.00201
500K	481	1.54	0.40	3.9899(21)	-0.048(6)	16.007	0.002232(10)	0.00221
600K	580	1.52	0.50	4.6081(20)	-1.012(5)	16.050	0.002398(20)	0.00245
700K	688	1.41	0.61	4.6236(19)	-0.885(5)	16.082	0.002710(20)	0.00274
800K	787	1.58	0.62	4.6471(22)	-0.792(5)	16.055	0.002960(10)	0.00302
900K	885	1.41	0.54	4.6502(20)	-0.744(5)	16.059	0.003160(21)	0.00331
1000K	973	1.44	0.56	4.6198(20)	-0.633(5)	16.097	0.003418(19)	0.00358

Table S6. Lattice constant and multipole parameters from *JANA 2006* of the final iteration of the WHCR procedure for the sample under air.

Sample name	Lattice const. *a* [Å]	κ_v	κ_v'	P_{32-}^*	P_{40}^*
100K	3.56692(1)	0.961(2)	0.951(8)	-0.305(4)	-0.118(8)
200K	3.56698(1)	0.944(2)	0.908(8)	-0.306(5)	-0.129(10)
300K-LT	3.56717(1)	0.949(2)	0.943(8)	-0.309(4)	-0.126(8)
300K-HT	3.56733(1)	0.966(2)	0.969(8)	-0.298(4)	-0.122(8)
400K	3.56783(1)	0.963(2)	0.960(8)	-0.298(4)	-0.131(8)
500K	3.56855(1)	0.958(2)	0.957(8)	-0.297(4)	-0.130(8)
600K	3.56977(1)	0.954(2)	0.954(8)	-0.291(4)	-0.131(8)
700K	3.57092(1)	0.963(2)	0.970(7)	-0.294(4)	-0.118(7)
800K	3.57228(1)	0.952(2)	0.943(7)	-0.317(4)	-0.150(8)
900K	3.57369(1)	0.948(2)	0.917(6)	-0.331(4)	-0.162(8)
1000K	3.57505(1)	0.951(2)	0.927(6)	-0.338(4)	-0.170(8)

Table S7. Peak profile parameters for the left-right split Thompson-Cox-Hastings pseudo-Voigt peak profile function from the final iteration of the Rietveld-Wilson procedure. *Parameter was fixed to the stated value.

Sample	Left					right				
name	U	V	W	LX	LY	U	V	W	LX	LY
100K	-1.57(17)	4.70(8)	0.172(11)	0.342(9)	3.11(6)	8.5(3)	2.2(2)	1.63(2)	0.313(9)	1.97(6)
200K	-2.82(16)	5.10(9)	0.113(11)	0.321(9)	3.24(6)	9.8(3)	1.7(2)	1.74(2)	0.307(9(	1.98(6)
300K-LT	-2.06(16)	4.29(8)	0.173(11)	0.307(9)	3.40(6)	8.5(3)	2.5(2)	1.69(2)	0.308(9)	2.03(6)
300K-HT	-1.61(21)	6.78(10)	0.100(12)	0.302(9)	2.32(7)	8.5(3)	2.4(2)	1.02(2)	0.383(9)	1.34(7)
400K	-2.02(21)	7.01(10)	0.032(12)	0.338(9)	2.15(7)	8.7(3)	2.5(2)	1.05(2)	0.39(1)	1.36(7)
500K	-2.67(21)	7.00(10)	0.054(12)	0.305(10)	2.35(7)	9.3(3)	2.3(2)	1.06(2)	0.38(1)	1.40(7)
600K	-4.82(22)	9.04(12)	0.441(15)	0.213(9)	3.22(6)	8.5(3)	-0.42(7)	0.538(8)	0.351(8)	2.65(6)
700K	-2.87(21)	7.55(11)	0.486(13)	0.228(8)	3.07(6)	7.4(2)	0.42(7)	0.505(8)	0.358(7)	2.55(5)
800K	-5.90(20)	8.28(12)	0.374(14)	0.189(9)	3.22(7)	11.2(3)	-0.72(9)	0.660(9)	0.343(8)	2.49(6)
900K	-3.11(19)	6.03(10)	0.579(12)	0.166(8)	3.31(6)	12.3(3)	-1.08(8)	0.765(9)	0.303(7)	2.58(5)
1000K	-1.90(20)	5.30(10)	0.588(12)	0.164(8)	3.29(6)	13.7(3)	-1.96(9)	0.995(9)	0.257(8)	2.66(6)

Supplementary Information References

Bansal, R. C., Vastola, F. J. & Walker Jr, P. L. (1972). *Carbon N. Y.* **10**, 443–448.

Barrer, R. M. (1936). *J. Chem. Soc.* 1261–1268.

Moelle, C., Klose, S., Szücs, F., Fecht, H. J., Johnston, C., Chalker, P. R. & Werner, M. (1997). *Diam. Relat. Mater.* **6**, 839–842.
